# Self-Consistent Estimation of Mislocated Fixations during Reading

**DOI:** 10.1371/journal.pone.0001534

**Published:** 2008-02-06

**Authors:** Ralf Engbert, Antje Nuthmann

**Affiliations:** Department of Psychology, University of Potsdam, Potsdam, Germany; Harvard Medical School, United States of America

## Abstract

During reading, we generate saccadic eye movements to move words into the center of the visual field for word processing. However, due to systematic and random errors in the oculomotor system, distributions of within-word landing positions are rather broad and show overlapping tails, which suggests that a fraction of fixations is mislocated and falls on words to the left or right of the selected target word. Here we propose a new procedure for the self-consistent estimation of the likelihood of mislocated fixations in normal reading. Our approach is based on iterative computation of the proportions of several types of oculomotor errors, the underlying probabilities for word-targeting, and corrected distributions of landing positions. We found that the average fraction of mislocated fixations ranges from about 10% to more than 30% depending on word length. These results show that fixation probabilities are strongly affected by oculomotor errors.

## Introduction

**Figure 1:**
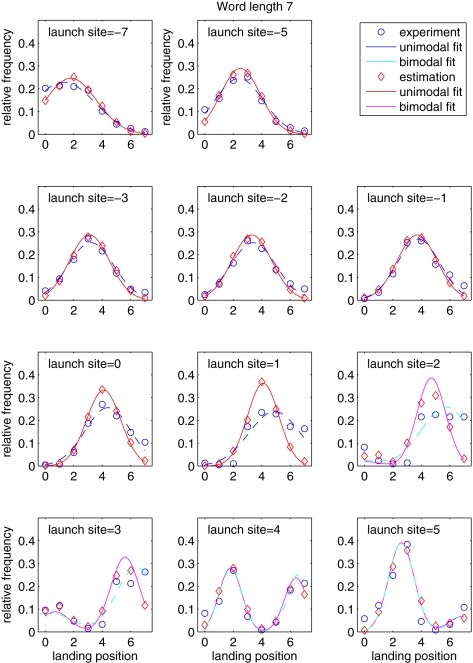
Distributions of within-word landing positions for words of length 7 (as an example). Letter position 0 is the space to the left of the word. Experimentally observed distributions (blue) are modulated by the distance of the launch site (the panels show distances between –7 and 5). Positive launch sites indicate refixations of the same word, which often lead to bimodal distributions (e.g., launch site = 3, 4). Estimated distributions (red) show a reduced standard deviation due to subtraction of mislocated fixations.

When you read these lines of text, you generate saccadic eye movements with an average rate of 3 to 4 per second [1] to enable efficient word processing in the center of the visual field (the fovea). Many words are skipped during reading, so that foveal processing is not necessary for all words, while some words need more than a single fixation, which causes refixations on the same word. For saccades, within-word landing positions (Fig. 1) show a pronounced peak near the word center [2], but distributions are rather broad and additionally modulated by word length as well as launch-site distance [3]. Landing position distributions can be approximated by normal distributions, however, these distributions are truncated at word boundaries, suggesting that some of the fixations observed experimentally on a particular word were in fact intended for an adjacent word [3]. Such fixations are mislocated due to saccadic errors.

Mislocated fixations pose an important problem for the analysis of eye-movement data in reading because of the possible decoupling of fixation location and attention [4]: In a dual-task paradigm which required a target-directed saccade in combination with a letter discrimination task, saccadic error pattern indicated that attention was more reliably directed to the cued target location than saccadic eye movements, because discrimination performance on cued target locations was better than performance on actual landing positions in trials with saccadic errors, where saccades failed to land on the cued target. This effect demonstrated that covert attention was more precisely directed to a cued target location than the saccade.

For eye movements in reading, this result implies that we might process a different word than the fixated word during a mislocated fixation. Following this argument, misguided saccades that undershot the intended target word could create parafoveal-on-foveal effects [5]. In such effects, properties of the upcoming word (e.g., word difficulty) modulate the fixation duration of the currently fixated word (for an overview see [6]). The quantitative contribution of mislocated fixations is, however, an unsolved research problem, because there is no straightforward technique to investigate mislocated fixations. First, due the complexity of scanpaths in reading [1], [7], mislocated fixations cannot be identified from subsequent corrective saccades, which might occur in response to mislocated fixations. Second, mislocated fixations are difficult to study under experimental control in the laboratory.

**Figure 2:**
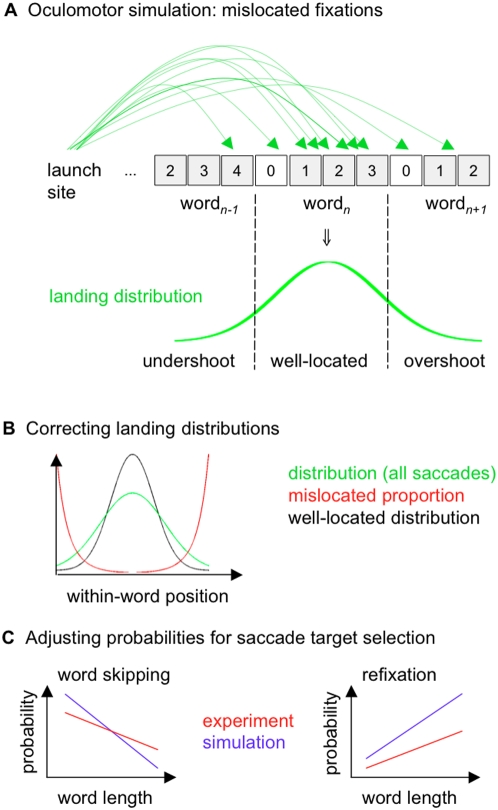
Iterative procedure for the estimation of mislocated fixations. Each iteration consists of three steps. (A) Oculomotor simulations are based on the parameters of the landing position distributions for a given launch site. Undershoot and overshoot of the target word generate mislocated fixations. (B) Landing position distributions are corrected by the amount of mislocated fixations as suggested by the simulations. (C) Mislocated fixations induce deviations from experimentally observed probabilities for word skippings (left) and refixations (right), which are adjusted after each iteration.

Here we propose a computational approach to the problem of mislocated fixations based on experimentally observed distributions of landing positions. The fraction of mislocated fixations can be estimated by extrapolation of experimentally observed landing distributions (Fig. 2a). The basic problem for such an approach is that experimental data of within-word fixation locations consist of both well-located (i.e., fixations intended for the realized target word) and mislocated fixations (i.e., fixations intended for adjacent words). The proportions of mislocated fixations as a function of within-word fixation position follows a U-shaped curve (Fig. 2b, red line) with higher probabilities of mislocated fixations near word boundaries [8] due to contributions from overlapping tails of the landing position distributions of adjacent words. We used numerical simulations of an oculomotor model (Fig. 2a) to estimate the proportion of mislocated fixations (see *Materials and Methods*). Simulations of this model permitted the direct computation of distributions of both mislocated and well-located fixations. Assuming that variance in landing positions is caused by oculomotor errors, we expect that distributions of well-located fixations (Fig. 2b, black line) show less variance than the original distributions of all fixations (green line), because the mislocated fractions near word boundaries are removed. A major complication for the estimation of the proportion of mislocated fixations is that these errors also bias probabilities for word skippings and refixations (Fig. 2c, red lines), such that simulated fixation probabilities deviate from the experimental data. As a solution to this problem, we developed an iterative procedure, where numerical simulations of saccade-targeting (Fig. 2a) were applied (i) to decompose the distributions of landing positions into well- and mislocated fixations (Fig. 2b) and (ii) to simultaneously adjust the probabilities for word-targeting, i.e., word skippings and refixations (Fig. 2c). Such an approach is self-consistent, because landing position distributions and word-targeting probabilities converge to numerical values consistent with self-generated errors.

The approach developed here represents a major improvement compared to our previously published work on mislocated fixations. In the first quantitative analysis of mislocated fixations, we estimated the proportions of mislocated fixation based on the assumption that each word (in serial order) is the target of a saccade [8]. Recently, we proposed an iterative procedure to compute the proportion a mislocated fixations from landing position data [9]. Both studies, however, were first-order approximations, because the (second-order) effect of mislocated fixations on fixation probabilities was neglected. For example, the general tendency to undershoot saccade targets will induce failed word skippings. As a consequence, any type of eye-movement model must produce a higher intended skipping rate than the skipping rate observed in the experiments to reproduce the experimental data. In the present self-consistent (and iterative) approach, we will adjust the intended fixation probabilities after each iteration step of our simulations to estimate proportions of mislocated fixations which are consistent with the observed patterns of fixation probabilities produced by the oculomotor system (Fig. 2).

## Materials and Methods

### Reading experiments

Eye-movement data from adult readers (*N* = 230; age range: 19 to 83 years) with normal or corrected-to-normal vision were recorded. Participants received study credit or were paid 5 €. Following ten practice sentences, all participants read the Potsdam Sentence Corpus [6], [10] comprising 144 single sentences and altogether 1138 words. Sentences were presented one at a time on a computer screen. The first and last fixations in a trial were excluded from the analyses. EyeLink I and II systems (SR Research, Osgoode, Canada) were used to measure a participant's gaze position with an absolute error of less than 0.5°, which corresponds approximately to letter size in our experiment. After preprocessing, data from 183,945 fixations were available. For more details on data preprocessing see [10].

### Oculomotor model

In a simple oculomotor model, we assumed that (i) fixation locations within words are drawn randomly from a word-length dependent landing-position distribution and that (ii) target words are selected according to a pre-defined set of word-length dependent probabililties for forward saccades, word skippings, and refixations. For simplicity, we excluded regressions. The model initially starts with the fixation probabilities found in our experimental data. However, due to saccadic errors from assumption (i), saccades realized in the model will not exactly follow the pattern in the experimental data. Therefore, we implemented a simulation approach with iterative update of intended fixation probabilities in the oculomotor model.

### Numerical simulations

For oculomotor simulations (Fig. 2a), parameters of (launch-site and word length contingent) distributions of within-word fixation positions were estimated using a grid search procedure. Distributions were fitted using truncated normal distributions (mean values and standard deviations were varied with a step size of 0.1 letter units). For launch sites close to the word center (e.g., Fig. 1, launch site = 3, 4), a bimodal fit was used to capture forward and backward refixation saccades (the same standard deviation was used for both saccade types). In our oculomotor model, the target word for each saccade was randomly selected according to the probabilities for word skipping and refixation as a function of word length. Next, the within-word landing position was drawn from the corresponding launch-site and word-length dependent distribution. For each sentence of the text corpus, *N* = 1000 runs were carried out to compute distributions of well-located and mislocated fixations positions (Fig. 2b), and the resulting fixation probabilities (Fig. 2c), as a function of word length. For each run, fixation probabilities were changed by half the deviation between simulated fixation probabilities and experimental values.

## Results

**Figure 3:**
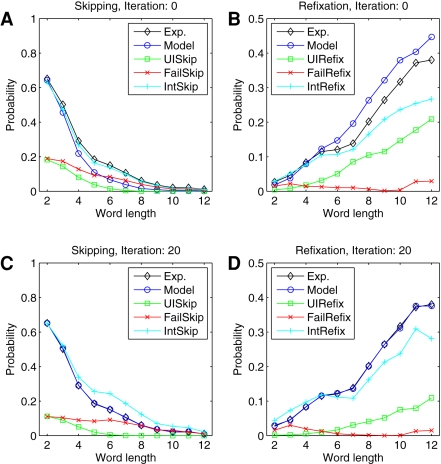
Saccade targeting under the influence of oculomotor errors. Mislocated fixations generate deviations between intended (light blue) and simulated (dark blue) fixation probabilities for word skippings (A,C) and refixations (B,D). After 20 iterations, probabilities for both types of saccades converged to the experimentally observed values (black). (A,B) Iteration 0. (C,D) Iteration 20.

The simulations started in step 0, where we used the experimentally observed skipping rates (Fig. 3a, black line) as the intended skipping rates (light blue). However, the numerical simulations showed that mislocated fixations strongly bias skipping probability as a function of word length in two ways. First, the realized saccade can undershoot the intended word (failed skipping) and, second, the saccade can overshoot the intended word (unintended skipping). In the simulations, failed skippings (Fig. 3a, red line) turned out to be more frequent than unintended skippings (green line). Therefore, over the full range of word lengths, realized skipping probabilities (dark blue line) are smaller than the values observed experimentally. To tackle this problem, our algorithm adjusted word-targeting probabilities in the next iteration, i.e., the algorithm increased the intended skipping probability for the next run of the oculomotor model.

This iterative procedure converged after about 20 steps (Fig. 3). Convergence showed that self-consistent estimation of mislocated fixations was successful. As a result, the probabilities for *intended* word skipping (Fig. 3c, light blue line) turned out to be higher than suggested by the experimental data (black line) for medium-sized word lengths. For long words (>6 characters), failed skipping is the most important error type. For decreasing word length, however, we found an increasing amount of unintended skippings. Because both errors types, i.e., unintended and failed skippings, are comparable in number for short words (<5 characters), the number of intended skippings equals the number of experimentally observed skippings.

**Figure 4:**
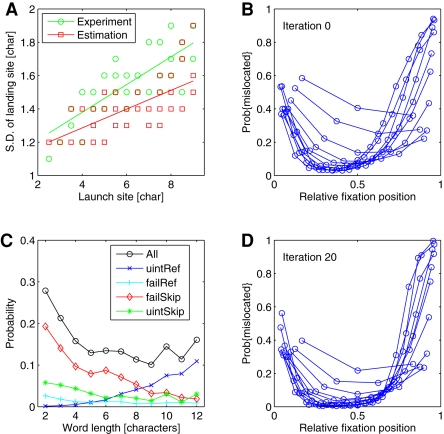
Random component of saccade errors and probabilities of mislocated fixations. (A) The random component of saccade errors is characterized by the standard deviation of the landing site distribution as a function of launch site distance. Distances are given as values from the *center* of the word. Best linear fits are also presented for experimental (green) vs. simulated (red) data. The reduced slope for the simulated data indicates the variance reduction achieved by our iterative estimation procedure. (B) Proportion of mislocated fixation as a function of relative fixation position for different word lengths (each curve represents a word length). For iteration 0, within-word distributions of mislocated fixations for word lengths 2 to 12 peak near word edges. (C) The overall probability of mislocated fixations (black) varies between about 10% and 30% for word lengths 2 to 12. For short to medium-sized words (<8 characters), failed skippings represent the most frequently occurring case of mislocated fixations, while for long words (>8 characters) unintended refixations are most important. (D) After 20 iterations, the proportion of mislocated fixations can be approximated by a single U-shaped curved for all words with lengths greater than 5 characters.

For refixations, a high rate of unintended refixations (Fig. 3b, green line) induced a higher refixation rate in the simulations than observed in the experimental data. After 20 iterations, however, the simulations suggested that the experimentally observed refixation probability (Fig. 3d, black line) can be decomposed into intended (light blue line) and unintended refixations (green line). Simultaneously to adjusting the fixation probabilities, our iterative procedure corrected the distributions of within-word landing positions by the amount of mislocated fixations (Fig. 1, red). As predicted, the resulting well-located landing position distributions are characterized by smaller variances (Fig. 2b). In the oculomotor model, this reduction of variance leads to a significantly reduced standard deviation of the random error component of saccades (Fig. 4a).

Given the experimental data (Fig. 1), we expected mislocated fixations to occur most frequently close to word edges. Iteration 0 was based on the assumption that all experimentally observed fixations are well-located. When landing position was normalized to one, we found that mislocated fixations occur frequently for relative landing positions smaller than 0.2 and/or for words consisting of less than 5 characters (Fig. 4b). Because our iterative procedure eliminated most of the variance of the curve across word lengths, proportions of mislocated fixations can be described by a single U-shaped curve for all word lengths (Fig. 4d) greater than three letters.

## Discussion

Our results indicate that the most important saccadic error types during reading are failed skippings and unintended refixations (Fig. 4c). Failed skippings represent the most prominent type of misguided saccades on short words, while unintended refixations typically fall on long words. Both targeting errors result from the eyes' general tendency to undershoot the center of target words [1]. This undershoot tendency increases with increasing launch site distance: The further away the launch site (i.e., the more negative the launch site distance for inter-word saccades), the more the mean of the Gaussian landing position distribution is shifted to the left (Fig. 1). This systematic linear component of oculomotor error, the so-called landing position function, has been explained in terms of a saccadic range error (SRE, [3]). The slope of the landing position function reflects the strength of the SRE. The observed experimental suggest a slope of 0.46, i.e., for each one-letter increment in (center-based) launch site distance, the mean of the landing position distribution is shifted by about half a letter. Different from this well-established finding [3], our simulations showed that this slope is reduced substantially to a value of 0.38 if empirical landing position data are corrected for mislocated fixations. Furthermore, we found a similar reduction of the slope from 0.63 to 0.45 for forward-refixation saccades. Thus, the present results are crucial for theoretical explanations of the landing position function in reading.

Why should our simple oculomotor model be adequate to investigate within-word landing positions and their impact on fixation probabilities in reading? The observation of Gaussian distributed fixation locations within words is a very robust phenomenon. There is somewhat controversial evidence as to whether cognition affects within-word landing positions. Based on well-controlled experiments, it was recently shown that orthographic familiarity and regularity influence landing positions [11]–[13]. In addition, earlier analyses of the present corpus reading data showed a small but significant effect of word frequency on mean landing site: Readers landed somewhat further into the word when it was a high-frequency word as compared to a low-frequency word; however, this was true for 3- to 6-letter words only [14]. Importantly, if observed at all, effects of higher-level cognitive variables on fixation locations are small (typically less than half a character). Furthermore, we recently compared normal reading data with data from a *z*-string scanning condition, conceptualized as an oculomotor control condition to normal reading. Landing position distributions were remarkably similar in both conditions [15]. We therefore conclude that oculomotor activity determining within-word fixation locations is largely independent from ongoing word processing during reading.

In a recent review of theories on word skipping [16], it was argued that “any comprehensive model of word skipping has to take into account the existence of involuntary word skipping due to oculomotor error (as well as the fact that some words are involuntary looked at because of a saccade undershoot)” (p. 60). Our present analyses contribute to this line of research by showing that unintended skippings due to saccadic overshoot are relatively rare (supporting an argument put forth by [17]). Rather, the impact of misguided saccades on skipping behavior predominantly shows as failed skippings, indicating that skipping probabilities as computed from experimentally observed data clearly underestimate the intended skipping probabilities, while the probability of intended refixations is overestimated from experimental data.

More generally, mislocated fixations represent an important source of error variance for conventional forms of analysis of eye-movement data. In perspective, our technique might be used to explain a significant proportion of this error variance in reading and other eye-movement tasks. Currently, there is an important debate on parafoveal-on-foveal in eye-movement research on reading. Because the word we are fixating on during a mislocated fixation may not necessarily be the word we are currently processing [5], parafoveal-on-foveal effects might partially be explained by mislocated fixations [18]. Our analysis proposed here is a promising approach to investigate the relation between mislocated fixation and parafoveal-on-foveal effects and to solve the controversy on parafoveal-on-foveal effects [19], [20].

Finally, mislocated fixations are a challenge for models of eye-movement control (e.g., [21]–[23]). Any model of saccade generation must reproduce the pattern of mislocated fixations described here. In the SWIFT model of eye-movement control (see [7] for the latest implementation of the model), we assumed that a mislocated fixation triggers the immediate start of a potentially error-correcting saccade program, which leads to reduced fixation durations for mislocated fixations. Such an assumption can explain the apparently paradoxical finding of an inverted-optimal viewing position effect in fixation durations [24]: An account based on visual acuity limitations would predict that fixation durations should be shortest around word centers, while experimentally fixation durations are longest at word word centers. Because mislocated fixations are more likely near word boundaries, the immediate start of a new saccade program generates the inverted U-shape of fixation duration as a function of within-word fixation location [8], [9], [15]. From this perspective, mislocated fixations represent a major factor influencing eye guidance during reading and related visual-cognitive behavior [25].
